# The impact of social-emotional learning: A meta-analysis in China

**DOI:** 10.3389/fpsyg.2022.1040522

**Published:** 2022-10-12

**Authors:** Huan Chen, Yanni Yu

**Affiliations:** Institute of Blue and Green Development, Shandong University, Weihai, China

**Keywords:** social-emotional learning (SEL), program assessment, uncertain social context, quantitative, social-emotional competence

## Abstract

The cross-cultural adaptation of social-emotional learning (SEL) has cast doubts. Although there are significant differences between low- and high-context cultures, few analyses have been conducted on the effects of SEL intervention in high-context cultures. To explore the effectiveness of the SEL program in China, which is different from low-context cultural background, this study presents findings from a meta-analysis of 86 randomized SEL programs involving 8,736 students. Compared with the control group, SEL participants significantly improved social-emotional competence (SEC), including SEL skills, attitudes, positive social behavior, and emotional distress (reduction). However, there was no significant improvement in behavioral problems. Due to the lack of emotional education in China and the Hawthorne effect, compared with SEL programs in low-context countries, China's SEL programs have improved SEC more, up to three times that of low-context countries. The general area of the school, SEL framework, intervention object, and educational level of participants moderates SEL positive outcomes. Types of textbooks, SEL framework, participant features, and educational level of participants moderate SEL negative outcomes. These findings provide empirical evidence for the positive impact of SEL programs in China. To improve the SEC of Chinese students, policymakers should actively implement SEL programs in China.

## Introduction

The OECD's answer to what kind of people society should produce in the face of future uncertainty is people with social and emotional competence (SEC). SEC is a set of core competencies related to self-management and social interaction, including the knowledge, identification, regulation, and expression of emotions (Denham, 2006). SEC is associated with a child's academic achievement, career preparation, and well being and is critical to personal success (Masten and Coatsworth, 1998; Guerra and Bradshaw, 2008; Alarcón Espinoza et al., 2022). The importance of SEC is also well-recognized in the field of education. At the OECD conference on “Skills for Social Progress” in 2014, education officials from 11 countries agreed that a balanced set of cognitive, social, and emotional skills would be essential to meet the challenges of the twenty-first century (OECD, 2015). In 2015, the “Education 2030 Action Framework” adopted by UNESCO put social-emotional learning on the global education policy agenda, proposing that students should master the social-emotional ability to deal with the various relationships between themselves and others, society, the country, and the world (UNESCO, 2015). Therefore, many countries have listed children's social-emotional learning (SEL) as national education content, and SEL has rapidly and widely diversified and been incorporated into schools and classrooms worldwide.

Numerous studies have established certain causal links between SEL programs and their participants' social and emotional skills, attitudes, behavior, and academic performance (Wilson et al., 2001; Durlak et al., 2011; Fernandez-Martin et al., 2021). Collaborative for Academic, Social and Emotional Learning (CASEL), the initiator of SEL, who has been leading the promotion and development of SEL, believes that the efficacy and effectiveness of SEL programs have cross-cultural adaptations. However, this cross-cultural adaptation of SEL has cast doubts. In the survey of SEL programs in Washington, D.C., many participants believed that unified adoption of given SEL standards and framework could not adapt to the local cultural background, but would become an obstacle to the further promotion of SEL programs (Petrokubi et al., 2019). Some researchers question whether SEL programs adequately reflect, cultivate, and leverage cultural assets and promote the well being of the youth of color and those from under-resourced backgrounds (Castro-Olivo, 2014; Jagers et al., 2018).

Compared with low-context cultures, such as the US and the UK, SEL faces more serious cultural adaptability problems when introduced into high-context cultures (Chong and Lee, 2015). Rooted in the past, high-context cultures are stable, unified, cohesive, and slow to change. Therefore, high-context cultures have close interpersonal relationships. Compared with the individualism of low-context culture, high-context culture is more inclined to collectivism (Nishimura et al., 2008). Some of the underlying principles in SEL, for example, were conceptualized from a cultural perspective that reinforces individualistic values of choice, personal responsibility, autonomy, and the importance of subjective experiences. This emphasis on individualism conflicts with high-context cultures, in which the family, community, and nation are considered to have greater importance than the self (Markus and Kitayama, 1991).

To study the effect of SEL programs in high-context cultures, this meta-analysis synthesizes the efficacy and effectiveness of SEL programs in China, a typical high-context country. This study presents a meta-analysis of 37 randomized SEL programs involving 8,736 students. Compared with the control group, SEL participants significantly improved SEC, including SEL skills, attitudes, positive social behavior, and emotional distress (reduction). But there was no significant improvement in behavioral problems. Due to the lack of emotional education in China and the Hawthorne effect, compared with SEL programs in low-context countries, China's SEL programs have improved SEC more, up to three times that of low-context countries. The general area of the school, SEL framework, intervention object, and educational level of participants moderates SEL positive outcomes. Types of textbooks, SEL framework, participant features, and educational level of participants moderate SEL negative outcomes. These findings provide empirical evidence for the positive impact of SEL programs in China. To improve the SEC of Chinese students, policymakers should actively implement SEL programs in China.

These findings in this paper also provide implications for other high-context cultures. Our study shows that SEL is effective in high-context culture countries, which indicates that SEL is a good start to promoting people's mental health in high-context culture countries. It is essential to take into account the different cultural contexts that contribute to mental health stigma when implementing SEL so that governments can better promote the effective implementation of SEL.

This paper contributes to the literature in three ways. First, we focused on China to study the effect of SEL in a high-context culture. Existing work mainly focuses on SEL conducted in low-context cultures. For example, Yang et al. (2019) evaluated the effects of the SEL program on the social-emotional outcomes of English language learners and found significant intervention effects. Although Durlak et al. (2011)'s meta-analysis did not restrict the countries where SEL intervention occurred, it was mainly the intervention of the United States. Although there are significant differences between low- and high-context cultures, there are few analyses on the effects of SEL intervention in high-context cultures. To fill in this gap, we study the effect of SEL in China.

Second, we overcame the disadvantages of traditional narrative literature review, adopted the meta-analysis method to obtain the conclusions of previous literature, and conducted a comprehensive study on the effect of SEL on this basis. Meta-analysis can provide objective quantitative standards and eliminate biases in the analysis process, to truly discover the relationship and strength between variables.

Third, from the study design, we analyzed the reasons for the inconsistent effects of SEL intervention. The object of SEL intervention, the way of intervention, and the time of intervention were inconsistent. For example, some interventions are for rural schools and some are for urban schools. Due to limitations in study design, it is difficult to include multiple interventions in a single study, resulting in inconsistent conclusions. We used meta-analysis to explore the moderating variables of SEL programs, and then analyze the key factors to ensure the success of SEL programs.

## SEL and SEL programs in China

Educators from all over the world have gradually realized that it is not conducive to the long-term development of students to emphasize cognitive training while neglecting the cultivation of students' confidence, self-esteem, and getting along well with others, which are necessary and critical qualities to promote their personal development and adapt to society. As a result, CASEL has launched an educational reform campaign, the SEL Program, which aims to make SEL a compulsory part of school education in all grades from kindergarten to high school, so that students can acquire this indispensable life skill for their success in school and the future (Fernandez-Martin et al., 2021). In 2015, the “Education 2030 Action Framework” adopted by UNESCO put SEL on the global education policy agenda, proposing that students should master the social-emotional ability to deal with the various relationships between themselves and others, society, the country, and the world (UNESCO, 2015). Therefore, many countries have listed children's SEL as the content of national education, and SEL has rapidly and widely diversified and been incorporated into schools and classrooms worldwide.

Taking 2011 as the cut-off point, we divided China's SEL intervention into two periods. First, as early as 2002, some Chinese scholars in psychology and education introduced the International SEL Program. These scholars introduced programs such as Promoting Alternative Thinking Strategies, Second Step, and Strong Kids to China. These programs benefited a limited number of children, mainly for research purposes. During this period, many training institutions, such as Buck tooth Rabbit Children Emotional Intelligence Park and Huigen Emotional Intelligence College, have begun to teach SEL as an independent course. However, compared with the comprehensive promotion and government support of SEL in some countries, China is still in the informal exploration stage of SEL.

Second, in 2011, the Ministry of Education and UNICEF launched a pilot SEL program in five counties of five provinces in western China, including Guizhou, Yunnan, Chongqing, Guangxi, and Xinjiang, with more than 250 schools participating as pilot schools. By 2016, the program had spread to 11 provinces in eastern, central, and western China, covering more than 500 primary and secondary schools with more than 200,000 students. Different from the theoretical and practical exploration of SEL programs in the first stage, the second stage, supported by the government, focuses more on the comprehensive education reform of SEL programs at the school level.

## Methods

### Literature search

#### Search strategies

This paper collects literature on the subject of SEL in China and uses four search strategies to collect all published Chinese and English literature. First, we use search terms for a comprehensive search. We use “social-emotional learning,” “social and emotional learning”, “social-emotional education,” “team counseling,” and “intervene” as keywords to perform searches in the China National Knowledge Infrastructure (CNKI) and collect related published literature; In addition, for English literature, we searched Pub Med, Springer Link, EBSCOhost, the Web of Science database with “social and emotional learning,” “social-emotional education” as the keywords. Second, we conducted manual searches to supplement the literature. We conducted manual searches on important journals that published literature related to social and emotional learning. These journals include Psychological development and education (in Chinese), Psychological Science (in Chinese), China Journal of Health Psychology (in Chinese), Chinese Journal of Clinical Psychology (in Chinese), Psychological Exploration (in Chinese), Acta Psychologica Sinica (in Chinese), Advances in Psychological Science (in Chinese), Chinese Journal of Applied Psychology (in Chinese). Third, manually searched the websites of social-emotional learning programs to screen for qualified research, such as the UNICEF official website, the “Social-emotional Learning” column of the UNICEF WeChat Official Account, and the “Social Emotional Learning” WeChat public account created by Beijing Normal University. Fourth, we manually searched the reference lists of the collected literature to avoid missing important literature. Since we are the first meta-analysis to study Chinese social and emotional learning, none of the studies we reviewed has been included in any previous review.

#### Inclusion and exclusion criteria

After collecting the literature, we reviewed the articles that met the following inclusion criteria: (a) being an intervention study with the control group; (b) appearing in published or unpublished form by July 30, 2022; (c) being conducted in China; (d) writing in English or Chinese;(e) reporting sufficient information to calculate the effect sizes (ESs); (f) all types of SEL interventions, including school, classroom and group-based intervention.

#### Selection procedure

**Figure 1:**
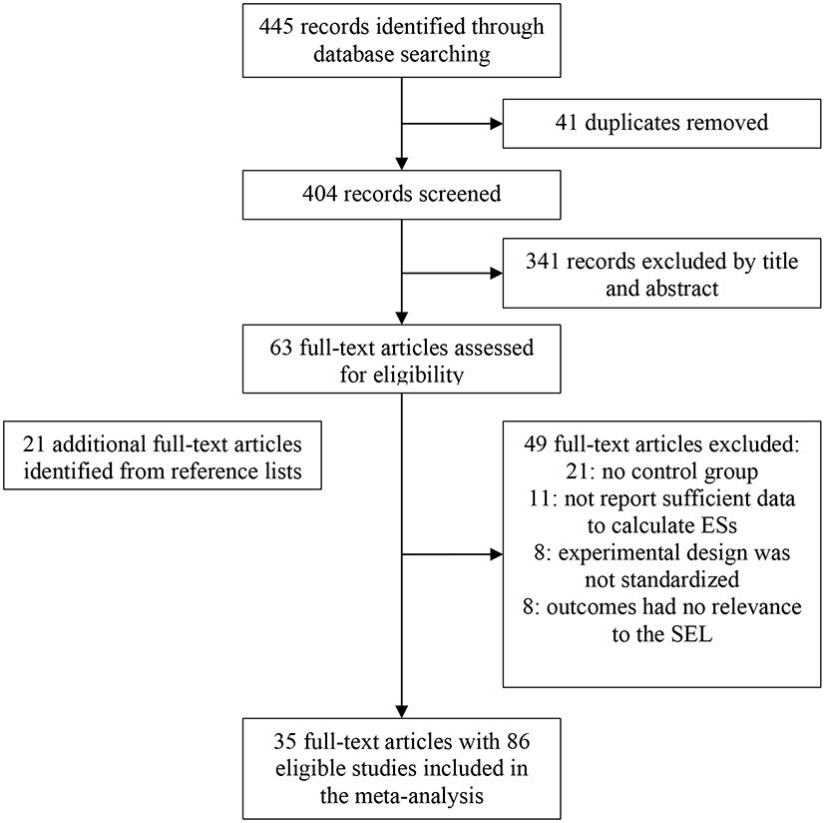
The selection process for studies included in the meta-analysis.

As shown in Figure 1, 445 reports were reached based on various databases, and 41 duplicate reports were removed. Three hundred and forty one were excluded according to title and abstract. The full text of the remaining 63 reports was screened in detail. We performed a manual search based on the reference lists of the 63 reports, and 21 more reports were added. Of the remaining 84 articles, we excluded another 49 studies for the following reasons: (a) 21 reports had no control group; (b) 11 reports did not report sufficient data to calculate ESs; (c) The experimental design of eight reports was not standardized; (d) The outcomes of eight reports had no relevance to the SEL.

### Data extraction

#### Independent variables

The independent variables included SEL interventions in China, which could be educational programs or curriculums that aimed to promote SEC of the student (Yang et al., 2019).

#### Dependent variables

We divide the outcomes for measuring SEC into positive outcomes and negative outcomes. The larger the positive outcomes are, the better the effect is. The smaller the negative outcomes are, the better the effect is.

Positive outcomes included: (a) SEL skills, including scores for various types of cognitive, affective, and social skills related to SEL (Durlak et al., 2011), such as creativity, interpersonal skills, cognitive skills, problem-solving skills, psychological resilience, adversity tolerance, and emotional regulation. SEL skills emphasize individual abilities, which can be reflected through questionnaires, scales, or performance on structured tasks. In contrast, attitudes emphasize individual attitudes, positive social behaviors emphasize individual behaviors. (b) Attitudes, including positive attitudes toward self, others, groups, and society, such as self-esteem, self-awareness, satisfaction with oneself, personal evaluation, liking of classmates, compassion for others, and belief in helping others. Attitudes emphasize personal attitudes, which are reflected by questionnaires or scales of students themselves. (c) Positive social behaviors, including adaptive or constructive behaviors, are used to deal with challenging social situations and reduce stress (Yang et al., 2019), such as seeking support from others and prosocial behaviors. Positive social behaviors emphasize individual behavior and are a daily occurrence rather than the performance of hypothetical situations.

Negative outcomes included: (a) Conduct problems, including different types of bad behavior, such as hyperactivity, disruptive class behavior, having trouble interacting with people, internet addiction, and aggression. Conduct problems are also a daily occurrence. (b) Emotional distress, including measures of various mental health problems, such as depression, anxiety, stress, fear, loneliness, and low self-esteem.

#### Potential moderators of outcomes

The moderators that affect SEL outcomes are diverse. We mainly investigated the research object and research design to analyze which people to carry out SEL and what kind of SEL design can achieve better results. Potential moderators included:

(a) General area of the school (Fernandez-Martin et al., 2021). The general area of the school might have affected SEL outcomes because the mental health of rural and urban students is unbalanced due to the different social and emotional concerns. The general area of the school is mainly divided into rural, suburban, and urban.
(b) Types of textbooks. The practice of SEL in China started late and there is no unified textbook, so different textbooks are used for academic exploration. Some studies applied textbooks from developed countries to guide Chinese students, such as the Strongkids Curriculum (Deli et al., 2021) and the PATHS Curriculum (Kam et al., 2011). From 2011 to 2020, UNICEF and the Ministry of Education introduced the SEL program, building a sinicized theoretical framework. Some studies have also applied this textbook to guide students (Wang et al., 2016). The above two applied published standard textbooks were labeled standard textbooks. And some researchers write textbooks to guide students according to the principles of SEL (Wong et al., 2014). If the research provides information about the textbooks, it was labeled self-compiled textbooks; if it only lists brief steps, it was labeled learning process; if it does not mention the textbooks, it was labeled did not report.
(c) SEL framework. After SEL was introduced into China, the Ministry of Education of China and UNICEF formed a sinicized SEL framework including self-cognition, self-management, cognition of others, management of others, cognition of the collective, and management of the collective based on the traditional Chinese cultural values and the reality of China's basic education (Yu and Jiang, 2017). Self-cognition is knowing about one's feelings, interests, values, and strengths. Self-management is adjusting one's own emotions and behaviors, regulating one's pressure, and stimulating one's own volition. Cognition of others is recognizing and understanding other people's attitudes, feelings, interests, perspectives, and behaviors. Management of others is establishing and maintaining friendly interpersonal relationships. Cognition of the collective is understanding the rules, norms, and values of the collective and its perspective, forming a sense of belonging and honor. Management of the collective is conforming to collective norms and adjusting the relationship between individual and collective. If the SEL intervention adopted in a study contains these six dimensions, it was labeled sinicized SEL framework; otherwise, it was labeled a non-sinicized SEL framework.
(d) Participant features. Some studies focus on students who had pre-existing behavioral and emotional problems such as internet addiction, anxiety, and poor mental health. For such a study, we coded the participant features as “problematic students.” And some of the studies were just randomly selected average students, and we coded the participant features of such studies as “ordinary students.”
(e) Intervention object. We coded the intervention object into two groups: for students only and also for parents. If a study only performed SEL intervention on students, the intervention object was labeled for students only. If a study supplemented the intervention with some guidance to the student's parents, the intervention object was labeled also for parents.
(f) Duration of intervention. Duration of intervention might have affected SEL outcomes (Yang et al., 2019). If the duration of intervention in a study is <600 min, the duration of intervention was labeled <600 min. If the duration of intervention in a study is ≥600 min, the duration of intervention was labeled ≥600 min.
(g) Educational level of participants (Fernandez-Martin et al., 2021). The educational level of participants might have affected SEL outcomes. SEL may be more effective for primary and secondary school students (Yang et al., 2019). We coded the educational level of participants into two groups: junior high and below, and high school and above.
(h) Intervention format. We coded the intervention format into two groups: Class by teacher and Class by non-school personnel (Fernandez-Martin et al., 2021). Some of the studies involved intervention by non-school personnel, such as researchers themselves or hired specialized psychologists, which we labeled Class by non-school personnel, and some of the studies involved intervention by teachers from schools that students were more familiar with, who were trained to perform SEL intervention according to the process, which we labeled class by teacher.
(i) Data collection time. We coded the data collection time into two groups: follow-up and immediate. If the outcomes were collected after the last intervention, we labeled them as immediate, and if the outcomes were followed up after the last intervention, we labeled them as a follow-up.

#### Coding reliability

After identifying 86 studies from 35 reports included in the meta-analyses, the first author established a coding system to record information about each study, e.g., study publication year, author, literature source, general area of the school, types of textbooks, SEL framework, participant features, intervention object, duration of intervention, educational level of participants, intervention format, data collection time, measured outcomes and sample size of experimental group and control group respectively. After identifying the data that should be extracted, we independently reviewed the studies by multiple evaluation teams composed of different personnel. Specifically, four researchers, including the first author, were divided into two groups for data extraction. The corrected Kappa coefficient is 0.80, indicating that the level of agreement is acceptable.

#### Computing and combining ESs

Cohen's d is the most widely used ES in meta-analysis. However, since this meta-analysis included some small sample studies, the ES was calculated using Hedge's g to correct the small sample bias (Hedges, 1981). Hedge's g was the index that represents the standardized difference between means. All ESs were calculated in such a way that positive values indicated a favorable result for the SEL intervention group over the control group. One ES per study was calculated for each outcome category. We used the overlapping CIs (Cumming and Finch, 2005) to determine whether the mean ESs from different studies differed significantly.

This meta-analysis used a random-effects model for all analyses. The fixed-effects model assumes that each independent study comes from the sample of the same population, and the effect value of each study is only a realization of the population parameter. The random-effects model refers to that each study comes from different populations, and each study has significant variability. The random-effects model tends to be conservative. Since the data for this meta-analysis were derived from a series of published studies conducted by different people, measuring different outcomes with different instruments, we used a random-effects model.

## Results

### Publication bias in reviewed studies

**Figure 2:**
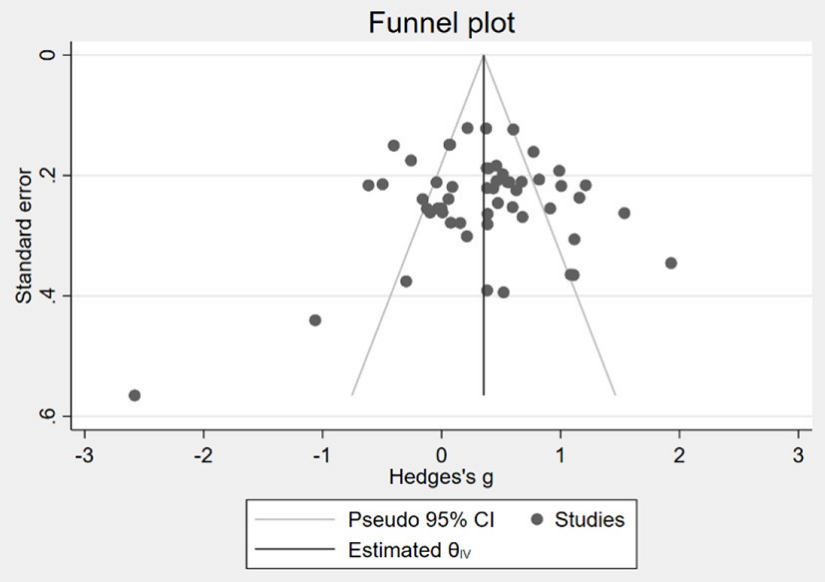
Funnel plot of publication bias in positive social-emotional performance.

**Figure 3:**
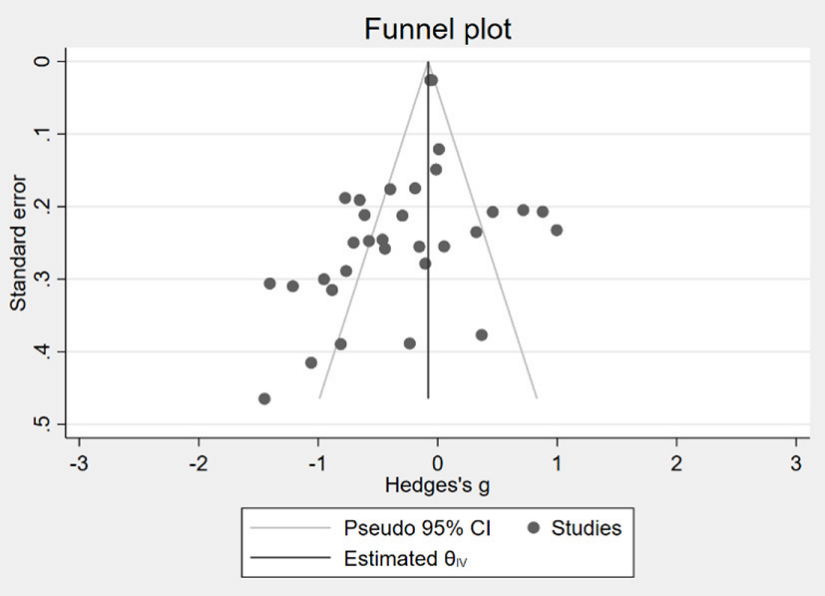
Funnel plot of publication bias in negative social-emotional performance.

Before the meta-analysis, this paper examines the presence of publication bias in the studies included in the meta-analysis. The funnel plot was first used to identify the publication bias of the study. Positive and negative outcomes were tested separately. The positive outcomes are shown in Figure 2, and the negative outcomes are shown in Figure 3. Both Figures 2, 3 show that scattered points are generally symmetric, indicating that little publication bias was detected.

Due to the inaccuracy of the funnel plot, the Egger regression and Macaskill regression methods were also adopted in this paper to further objectively test publication bias. Table 1 reports the results of the two publication bias tests. Columns (1) and (2) are tests of positive outcomes, while columns (3) and (4) are tests of negative outcomes. Columns (1) and (3) are Egger regression. The coefficient of std. err. in column (1) is insignificant, indicating no publication bias in the sample of positive outcomes, while the coefficient of std. err. in column (3) is significant, indicating that there is publication bias in the sample of negative outcomes. Columns (2) and (4) are Macaskill regression. The coefficients of sample size in columns (2) and (4) are insignificant, indicating no publication bias in the samples.

**Table 1:** The results of Egger regression and Macaskill regression.

**Dep. Var**.	**Positive outcome**		**Negative outcome**	
	**ES (1)**	**ES (2)**	**ES (3)**	**ES (4)**
Std.err.	−1.340 (1.043)		−2.553** (1.044)	
Sample size		0.001 (0.002)		0.000 (0.000)
Constant	0.679*** (0.260)	0.254 (0.172)	0.309 (0.264)	−0.351 (0.116)
Observations	54	54	32	32

***p < 0.01, **p < 0.05.

Since negative outcomes have some publication bias, we use the trim and fill method (Duval and Tweedie, 2000) to correct them. The trim and fill analyses resulted in no change in the estimated mean effects. All of the estimated means from the trim and fill analyses remained significantly different from zero. Based on the above test methods, we can conclude that there is no conclusion of publication bias in the studies included in the meta-analysis.

### Descriptive characteristics of reviewed studies

A total of 86 intervention studies from 35 reports were analyzed. There were approximately 8,736 children involved. Table 2 summarizes the general features of these SEL Interventions. The first SEL intervention we found in China was in 2003, and there have been more and more since then. More than half of SEL interventions were published between 2013 and 2021 (56%). Most SEL interventions were conducted in urban schools (85%) and 9% in suburban schools, with the least amount of interventions in rural schools (6%). More than half of the studies reported learning process (60%), and some used self-compiled (15%) or standardized textbooks (19%). More than half of the studies involved the intervention with the Sinicized SEL framework (58%). Most interventions are aimed at ordinary students (76%), with the remainder aimed at problematic students (24%). Most interventions are for students only (84%), and a small number of interventions also target parents (16%). More than half of the interventions lasted more than 600 min (58%). More than half of the interventions were for students in junior high school and below (59%). Most interventions were classed by non-school personnel (81%). More than half of the interventions collected data immediately (64%).

**Table 2:** Descriptive characteristics of 86 SEL interventions.

**General features**	**N**	**%**
**Date of report**
2003–2012	38	44.19
2013–2021	48	55.81
**General area of the school**
Rural	5	5.81
Suburban	8	9.30
Urban	73	84.88
**Types of textbooks**
Did not report	5	5.81
Learning process	52	60.47
Self-compiled textbooks	13	15.12
Standardized textbooks	16	18.60
**SEL framework**
Non-sinicized SEL framework	36	41.86
Sinicized SEL framework	50	58.14
**Participant features**
Problematic students	21	24.42
Ordinary students	65	75.58
**Intervention object**
Also for parents	14	16.28
For Students only	72	83.72
**Duration of intervention**
< 600 min	36	41.86
≥600 min	50	58.14
**Educational level of participants**
Junior high and below	51	59.30
High school and above	35	40.70
**Intervention format**
Class by Non-school personnel	70	81.40
Class by teacher	16	18.60
**Data collection time**
Follow-up	31	36.05
Immediate	55	63.95

### Effects of the SEL

We classified the ESs by positive and negative outcomes. Table 3 shows the mean ESs for the positive outcomes of SEL interventions was 0.361 (CI = 0.211–0.512), which was statistically significant from zero. And the mean ESs for the negative outcomes of SEL interventions was −0.292 (CI = −0.499–−0.085), which was statistically significant from zero. All in all, SEL interventions enhance SEC in China.

**Table 3:** The mean effects and heterogeneity tests of SEL.

**Variable**	**N**	**Mean ES**	**95% CI**		**Z Sig**.	**Q**	**I^2^**
			**Lower**	**Upper**			
**Positive outcomes**	54	0.361	0.211	0.512	4.70***	280.79***	84.74%
SEL skills	25	0.361	0.098	0.652	2.69***	156.86***	88.48%
Attitudes	13	0.334	0.038	0.629	2.21***	56.97***	83.22%
Positive social behavior	16	0.372	0.137	0.608	3.10***	66.77***	80.35%
**Negative outcomes**	32	−0.292	−0.499	−0.085	−2.77***	190.99***	95.61%
Conduct problems	12	−0.348	−0.730	0.033	−1.79*	68.31***	91.31%
Emotional distress	20	−0.265	−0.515	−0.014	−2.07**	122.63***	91.25%

***p < 0.01, **p < 0.05, *p < 0.1.

In terms of specific outcomes, SEL interventions had significant effects in improving SEL skills (mean ES = 0.361, *p* < 0.01), attitudes (mean ES = 0.334, *p* < 0.01), positive social behavior (mean ES = 0.372, *p* < 0.01), and significantly reduced emotional distress (mean ES = −0.265, *p* < 0.05). However, the 95% confidence interval of conduct problems contains zero, indicating that the effect value has no statistically significant difference from zero, indicating that SEL intervention has no significant effect on conduct problems.

We also examine the significance of the heterogeneity of ESs. The Q statistic of each outcome was significant, indicating that there was considerable heterogeneity in the ESs of studies. In addition to the Q statistic, we also use I2, because the Q statistic can be biased when the number of studies is small. I2 lies between 0 and 100%. A value of 0% indicates no observed heterogeneity, and larger values show increasing heterogeneity. Higgins et al. (2003) divided heterogeneity into low, medium, and high levels by 25, 50, and 75%, respectively. I2 of each outcome is >75%, indicating high heterogeneity, which means that the fixed-effects model is not appropriate. Therefore, we calculated the average ESs using the random-effects model, and we need to further analyze the source of ESs heterogeneity.

### Moderator analyses

We first examined the potential moderators using subgroup analyses. Table 4 shows the results of the subgroup analysis for the positive outcomes of SEL interventions, and Table 5 shows the results of the subgroup analysis for the negative outcomes. Subgroup analyses were grouped according to moderators, and Q statistical tests were performed to determine whether the ES of each group was equal. If the Q value is statistically significant (when *P* ≧ 0.01), it indicates heterogeneity among ESs.

**Table 4:** Subgroup analyses of ESs for potential moderators (positive outcomes).

**Potential moderators**	**Q**	**N**	**Mean ES**	**95% CI**		**I^2^**
				**Lower**	**Upper**	
**General area of the school**
Within group: Rural	3.10*	2	1.51	0.71	2.30	67.78%
Within group: Suburban	8.85*	5	0.47	0.26	0.67	54.81%
Within group: Urban	241.61***	47	0.30	0.14	0.46	82.81%
Between-groups heterogeneity	9.30***
**Types of textbooks**
Within group: Did not report	0.00	1	0.21	−0.38	0.80	.
Within group: Learning process	170.82***	34	0.47	0.26	0.68	87.22%
Within group: Self-compiled textbooks	46.23***	10	0.07	−0.21	0.34	82.23%
Within group: Standardized textbooks	22.14***	9	0.30	0.03	0.58	63.33%
Between-groups heterogeneity	5.46
**SEL framework**
Within group: Non-sinicized SEL framework	113.75***	21	0.06	−0.20	0.31	86.36%
Within group: Sinicized SEL framework	119.80***	33	0.55	0.38	0.71	77.78%
Between-groups heterogeneity	9.99***
**Participant features**
Within group: Problematic students	38.20***	11	0.50	0.14	0.86	75.66%
Within group: Ordinary students	240.35***	43	0.33	0.16	0.50	86.16%
Between-groups heterogeneity	0.71
**Intervention object**
Within group: Also for parents	15.00***	8	0.72	0.51	0.94	53.26%
Within group: For students only	239.60***	46	0.29	0.12	1.46	85.51%
Between-groups heterogeneity	9.47***
**Duration of intervention**
Within group: <600 min	118.98***	25	0.49	0.32	0.67	81.22%
Within group: ≥600 min	147.81***	29	0.23	−0.02	0.48	85.42%
Between-groups heterogeneity	2.92*
**Educational level of participants**
Within group: Junior high and below	38.20***	33	0.54	0.39	0.69	75.66%
Within group: High school and above	240.35***	21	0.06	−0.22	0.34	84.74%
Between-groups heterogeneity	8.50***
**Intervention format**
Within group: Class by non-school personnel	235.76***	44	0.35	0.17	0.54	86.27%
Within group: Class by teacher	43.43***	10	0.36	0.12	0.61	76.61%
Between-groups heterogeneity	0.00
**Data collection time**
Within group: Follow-up	104.22***	19	0.39	0.12	0.66	84.27%
Within group: Immediate	280.79***	35	0.35	0.17	0.53	84.74%
Between-groups heterogeneity	0.06

***p < 0.01, *p < 0.1.

**Table 5:** Subgroup analyses of ESs for potential moderators (negative outcomes).

**Potential moderators**	**Q**	**N**	**Mean ES**	**95% CI**		**I^2^**
				**Lower**	**Upper**	
**General area of the school**						
Within group: Rural	14.05***	3	−0.38	−1.07	0.31	99.62%
Within group: Suburban	6.30**	3	−0.29	−0.68	0.10	65.66%
Within group: Urban	163.06***	26	−0.28	−0.53	−0.03	85.87%
Between-groups heterogeneity	0.07
**Types of textbooks**						
Within group: Did not report	6.05	4	−0.83	−1.22	−0.44	50.70%
Within group: Learning process	117.73***	18	0.30	−0.60	0.01	87.60%
Within group: Self-compiled textbooks	5.46*	3	0.28	−0.63	0.07	62.48%
Within group: Standardized textbooks	27.00***	7	0.30	−0.36	0.35	98.04%
Between-groups heterogeneity	9.70**
**SEL framework**						
Within group: Non-sinicized SEL framework	42.97***	15	−0.51	−0.75	−0.28	69.85%
Within group: Sinicized SEL framework	118.80***	17	−0.09	−0.39	0.21	97.66%
Between-groups heterogeneity	4.87**
**Participant features**						
Within group: Problematic students	20.44**	10	−0.73	−1.04	−0.43	56.96%
Within group: Ordinary students	125.51***	22	−0.12	−0.35	0.11	96.19%
Between-groups heterogeneity	10.15***
**Intervention object**						
Within group: Also for parents	54.69***	6	−0.30	−0.92	0.32	91.33%
Within group: For students only	134.86***	26	−0.29	−0.51	−0.07	95.59%
Between-groups heterogeneity	0.00
**Duration of intervention**						
Within group: <600 min	59.51***	11	−0.12	−0.44	0.20	85.20%
Within group: ≥600 min	131.43***	21	−0.39	−0.66	−0.12	96.88%
Between-groups heterogeneity	1.59
**Educational level of participants**						
Within group: Junior high and below	126.56***	18	−0.13	−0.43	0.17	97.72%
Within group: High school and above	37.97***	14	−0.49	−0.72	0.26	67.08%
Between-groups heterogeneity	3.47**
**Intervention format**						
Within group: Class by non-school personnel	163.66***	26	−0.32	−0.57	−0.07	86.20%
Within group: Class by teacher	18.58***	6	−0.18	−0.48	0.12	97.35%
Between-groups heterogeneity	0.46*
**Data collection time**						
Within group: Follow-up	84.33***	12	−0.38	−0.73	−0.02	98.13%
Within group: Immediate	104.78***	20	−0.24	−0.50	0.02	84.99%
Between-groups heterogeneity	0.36*

***p < 0.01, **p < 0.05, *p < 0.1.

Table 4 shows that the general area of the school, SEL framework, intervention object, duration of intervention, and educational level of participants moderates SEL positive outcomes, as the Q statistics of between-groups are significant. And types of textbooks, participant features, intervention format, and data collection time do not moderate SEL outcomes. Specifically, the combined ES showed that interventions in rural areas might have positively affected outcomes for children's SEC (ES = 1.51), a significantly different finding from the effects of interventions in suburban areas (ES = 0.47) and interventions in urban areas (ES = 0.30). The possible reason is that the SEC of children in rural areas is lower because of elevated poverty rates, limited access to public transportation, difficulty retaining qualified personnel, and the cultural stigma associated with mental health support (Mitchell, 2021). As for the types of textbooks, although there is no significant heterogeneity, it can be seen that those with standardized textbooks (ES = 0.30) and learning processes (ES = 0.47) are more significant. Studies with sinicized SEL framework might have had significantly stronger effects in enhancing children's positive social-emotional outcomes (ES = 0.55) in China. SEL interventions work for both problematic students (ES = 0.50) and ordinary students (ES = 0.33). SEL intervention also for parents can achieve better results (ES = 0.72). Because parental involvement is good for SEC (Roy and Giraldo-García, 2018). SEL intervention is better for students in junior school and below (ES = 0.54). This is also consistent with previous research (Yeager, 2017). The effect was similar whether the SEL intervention was classed by teachers or non-school personnel. The effect was similar whether the data were collected immediately after the intervention or at follow-up. As for the result duration of intervention, the effect was smaller when the time was longer than 600 min, which was not consistent with our expectations. We further studied it with meta-regression analysis.

Table 5 shows that types of textbooks, SEL framework, participant features, and educational level of participants moderates SEL negative outcomes. Specifically, the studies that did not report textbooks achieved more significant results (SE = −0.83). Similarly, Studies without sinicized SEL framework also achieved more significant results (SE = −0.51). This is different from the positive outcomes. According to the data, this is because there are some SEL interventions specifically for problematic students in the study, which do not report textbooks and do not conform to the Sinicized SEL framework, but they are very effective in improving conduct problems and emotional distress. Accordingly, these SEL interventions have achieved better results for problematic students (SE = −0.73). Another difference between the negative and the positive outcomes is that the improvement in the negative outcomes is more effective for High school and above students (SE = −0.49). It could be that older students have more conduct problems and emotional distress.

To further analyze the effect of study design on ESs, this paper also uses the meta-regression method to verify. Table 6 presents the results of the meta-regression analysis based on a random-effects model. Column (1) is the result of the positive outcome, and column (2) is the result of the negative outcome. Table 6 shows that when other potential moderators are controlled and the duration of the intervention is controlled, the more the number of interventions, the better the effect on the positive outcome, while the effect on the negative outcome is not significant.

**Table 6:** The meta-regression of ESs for potential moderators.

**Potential moderators**	**Positive outcomes**	**Negative outcomes**
Number of interventions	0.025* (0.013)	0.022 (0.021)
Duration of intervention	0.000 (0.000)	−0.001 (0.000)
Control	Yes	Yes
Observations	54	32
*R* ^2^	0.46	0.13

***p < 0.01, **p < 0.05, *p < 0.1.

### Comparing ESs in different contexts

Next, we compare ESs in this study with ESs in low-context cultural contexts, to find out whether the effect of SEL intervention in China is consistent with that in low-context cultural contexts. Table 7 presents the overall mean ESs in this study, as well as similar results obtained from other SEL meta-analyses. Table 7 shows that the ESs of almost all studies in low-context cultural contexts were lower than ours, except that the ES of Durlak et al. (2011)'s SEL skills was higher than ours, which indicated that China's SEL intervention had achieved considerable effects, which were greater than the effect in low-context cultural contexts, and the maximum is three times that of low-context cultural contexts.

**Table 7:** Comparing ESs to previous meta-analyses in low-context cultural contexts.

**Outcomes**	**Mean ESs**		
	**This meta-analysis**	**Other meta-analyses**	
**Positive outcomes**	0.361	0.57	Durlak et al., 2011
		0.183	Yang et al., 2019
SEL skills	0.361	0.34	Murano et al., 2020
		0.23	Taylor et al., 2017
		0.12	Yang et al., 2019
		0.23	Durlak et al., 2011
Attitudes	0.334	0.13	Taylor et al., 2017
		0.24	Durlak et al., 2011
Positive social behavior	0.372	0.13	Taylor et al., 2017
		0.20	Yang et al., 2019
		0.22	Durlak et al., 2011
**Negative outcomes**	−0.292	−0.10	Yang et al., 2019
Emotional distress	−0.265	−0.16	Taylor et al., 2017
		−0.24	Durlak et al., 2011

In order to compare with the results of this meta-analysis, we change the sign of the negative results of other meta-analyses.

There may be two reasons for this. One is the lack of emotional education in China. For a long time in China, education has been influenced by utilitarian thought. People excessively care about and pursue the cognitive level of human wisdom and obvious test scores, but turn a blind eye to the lack of emotional education. Such education leads to different degrees of psychological and behavioral problems among students. Studies show that in China, primary school students with psychological and behavioral problems account for about 10% of the total (Yang and Mao, 2017). Therefore, in this environment, SEL can achieve better results. The second reason is the nature of SEL interventions in China. Most SEL interventions in China are small-scale, experimental studies designed to test the effectiveness of programs developed by researchers, rather than widely promoted programs. Therefore, these specific experimental environments may result in better effects than expected. This is also known as the “Hawthorne effect”— the experimental group may show more positive behaviors than expected due to the influence of the experimental environment (Merrett, 2006). However, even though greater ESs may be obtained due to the “Hawthorne effect,” we can definitely conclude that SEL interventions are still effective in high-context cultural contexts.

## Conclusion

The cross-cultural adaptation of SEL has cast doubts. To explore the effectiveness of the SEL program in China, which is different from low-context cultural background, this study presents findings from a meta-analysis of 86 randomized SEL programs involving 8,736 students.

The main conclusions of this paper include: First, SEL interventions enhance SEC in China. SEL interventions had significant effects in improving SEL skills (mean ES = 0.361, *p* < 0.01), attitudes (mean ES = 0.334, *p* < 0.01), positive social behavior (mean ES = 0.372, *p* < 0.01), and significantly reduced emotional distress (mean ES = −0.265, *p* < 0.05). However, SEL intervention has no significant effect on conduct problems.

Second, subgroup analysis finds that the general area of the school, SEL framework, intervention object, and educational level of participants moderates SEL positive outcomes. Types of textbooks, SEL framework, participant features, and educational level of participants moderate SEL negative outcomes.

Third, the meta-regression finds that when other potential moderators are controlled and the duration of the intervention is controlled, the more the number of interventions, the better the effect on the positive outcome, while the effect on the negative outcome is not significant.

Fourth, compared with SEL programs in low-context countries, China's SEL programs have improved SEC more, up to three times that of low-context countries.

These findings in this paper provide important policy notes for the implementation of SEL programs in China. First, The SEL programs with sinicized SEL framework and standardized textbooks can achieve better results. Therefore, the implementation of SEL in China can follow the social-emotional learning teaching book compiled by the Ministry of Education and UNICEF, because it is a standardized textbook and adopts sinicized SEL framework.

Second, The SEL programs significantly improve the positive SEL outcomes, but the negative SEL outcomes are not ideal. Therefore, more targeted psychological interventions are needed for negative SEL outcomes, such as behavioral problems.

Third, SEL programs work better in rural areas. Therefore, we should further promote the implementation of SEL programs in rural areas. In addition, SEL programs with parents' participation have better effects, so we should actively promote the participation of parents.

Fourth, in the course arrangement, when the duration of intervention is fixed, the more intervention times, the better the effect. Therefore, when conducting SEL programs, we should arrange more interventions to achieve better results.

These findings in this paper also provide implications for other high-context cultures. Cultural differences shape societies' attitudes toward mental health. Compared with low-context cultures, people in high-context cultures believe that depression brings shame to the family (Ng and Li, 2010). As a result, high-context culture countries do not pay enough attention to psychological education. Our study shows that SEL is effective in high-context culture countries, which indicates that SEL is a good start to promoting the mental health of people in high-context culture countries. It is important to take into account the different cultural contexts that contribute to mental health stigma when implementing SEL so that governments can better promote the effective implementation of SEL.

## Data availability statement

The raw data supporting the conclusions of this article will be made available by the authors, without undue reservation.

## Author contributions

All authors contributed to all aspects of this work-design, analysis, and writing and approved the submitted version.

## Funding

This research was supported by National Natural Science Foundation of China (Grant number: 72022009).

## Conflict of interest

The authors declare that the research was conducted in the absence of any commercial or financial relationships that could be construed as a potential conflict of interest.

## Publisher's note

All claims expressed in this article are solely those of the authors and do not necessarily represent those of their affiliated organizations, or those of the publisher, the editors and the reviewers. Any product that may be evaluated in this article, or claim that may be made by its manufacturer, is not guaranteed or endorsed by the publisher.
